# Hidden diversity of Acoelomorpha revealed through metabarcoding

**DOI:** 10.1098/rsbl.2016.0674

**Published:** 2016-09

**Authors:** Alicia S. Arroyo, David López-Escardó, Colomban de Vargas, Iñaki Ruiz-Trillo

**Affiliations:** 1 Institut de Biologia Evolutiva (CSIC-Universitat Pompeu Fabra), Passeig Marítim de la Barceloneta, 37-49, 08003 Barcelona, Spain; 2 CNRS, UMR 7144, Station Biologique de Roscoff, Place Georges Teissier, 29680 Roscoff, France; 3 Sorbonne Universités, Université Pierre et Marie Curie (UPMC) Paris 06, UMR 7144, Station Biologique de Roscoff, Place Georges Teissier, 29680 Roscoff, France; 4 Departament de Genètica, Microbiologia i Estadística, Universitat de Barcelona, Avinguda Diagonal 643, 08028 Barcelona, Spain; 5 ICREA, Pg. Lluís Companys 23, 08010 Barcelona, Spain

**Keywords:** Xenoacoelomorpha, metabarcoding, molecular diversity, origins of Bilateria, acoels

## Abstract

Animals with bilateral symmetry comprise the majority of the described species within Metazoa. However, the nature of the first bilaterian animal remains unknown. As most recent molecular phylogenies point to Xenacoelomorpha as the sister group to the rest of Bilateria, understanding their biology, ecology and diversity is key to reconstructing the nature of the last common bilaterian ancestor (Urbilateria). To date, sampling efforts have focused mainly on coastal areas, leaving potential gaps in our understanding of the full diversity of xenacoelomorphs. We therefore analysed 18S rDNA metabarcoding data from three marine projects covering benthic and pelagic habitats worldwide. Our results show that acoels have a greater richness in planktonic environments than previously described. Interestingly, we also identified a putative novel clade of acoels in the deep benthos that branches as sister group to the rest of Acoela, thus representing the earliest-branching acoel clade. Our data highlight deep-sea environments as an ideal habitat to sample acoels with key phylogenetic positions, which might be useful for reconstructing the early evolution of Bilateria.

## 1. Introduction

The vast majority of the described animal species are bilaterally symmetrical [1]. The establishment of two orthogonal body axes provided the basis for enormous structural complexity compared with radially symmetrical animals, which allowed a more diverse evolutionary outcome [2]. However, how bilaterians evolved and the nature of the first bilaterian animal remains elusive.

Bilaterian animals are separated into four major groups: Acoelomorpha, Ecdysozoa, Lophotrochozoa (or Spiralia) and Deuterostomia [1,3,4]. Although there has been some disagreement, it now seems clear that Xenacoelomorpha is the sister group to the rest of Bilateria (also known as Nephrozoa [5]) [6–8]. Thus, Xenacoelomorpha is a key taxon to compare with the rest of the bilaterians and reconstruct the nature of the last bilaterian common ancestor, namely Urbilateria.

Members of Xenacoelomorpha, which is formed by Acoela, Nemertodermatida and *Xenoturbella*, are morphologically quite simple: the digestive system only has one opening, they lack circulatory, respiratory and excretory systems, and also lack a body cavity between the gut and the epidermis [8,9]. Xenacoelomorphs live in benthic habitats, and the majority of described species have come from sediments, mainly in coastal areas [10–12]. This morphological simplicity of Xenacoelomorpha seems to support the planuloid–acoeloid hypothesis proposed by Von Graff [13] and Hyman [14], which envisaged Urbilateria to be a simple, benthic acoelomate organism exhibiting direct development [2,15].

However, the full diversity and morphological disparity of Xenacoelomorpha is not yet known, because it has never been approached in a systematic, high-throughput manner. It is therefore possible that there are unobserved or unsampled xenacoelomorph lineages with more complex morphologies or lifestyles, in different habitats, or occupying earlier phylogenetic positions in the Xenacoelomorpha tree. For example, some studies have described acoel morphospecies in freshwater [16,17], brackish water [18] and planktonic habitats [19]. Thus, any attempt to understand the nature and ecology of Urbilateria will require a more global and systematic analyses of Xenacoelomorpha diversity.

## 2. Material and methods

Clustered operational taxonomic units (OTUs) were obtained from public repositories or directly from the authors. The reference tree was constructed from 255 acoelomorph 18S rDNA GenBank sequences (from herein RefTree). Alignment was carried out using the E-INS-I option from MAFFT v. 7.271 [20] and manually trimmed. The maximum-likelihood (ML) tree was built using RAxML v. 8.0.0 [21] considering a GTR-GAMMA substitution model. Nodal support was obtained through 1000 bootstrap replicates. We selected the OTUs through RAxML-EPA [22] and chose those whose abundance was greater than 10 reads.

A final ML tree using both the RefTree sequences and our OTUs was inferred using RAxML [21], with the same conditions as above. A Bayesian tree was built using MrBayes v. 3.2.6 [23] using a GTR + I + *Γ* model of evolution. Pplacer v. 1.1 [24] was used to perform a phylogenetic placement of the OTUs into the RefTree. Novelty blast percentages were obtained running a blastn 2.2.31 [25] against our curated Acoelomorpha-GenBank database.

A more detailed description of Materials and Methods can be found in the electronic supplementary material.

## 3. Results and conclusion

**Figure 1.**
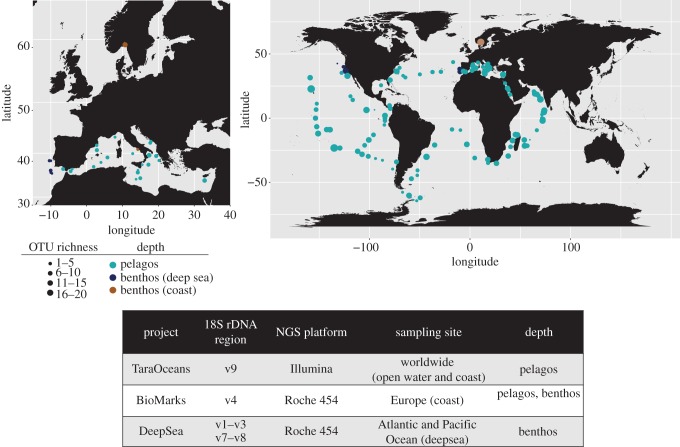
Worldwide distribution of Xenacoelomorpha OTUs. Top: distribution of Acoelomorpha across sampling sites and depth. Bottom: sequencing platforms and sampling information for the projects where the data were collected.

Here, we use a comprehensive metabarcoding approach with 18S rDNA to assess xenacoelomorph diversity in marine environments. The aim was to search for potential novel lineages that may be of interest to understand the ancestral xenacoelomorph body plan, as well as to identify the environments in which it would be possible to find them. To this end, we analysed the most complete marine eukaryotic metabarcoding datasets to date, comprising both benthic and pelagic marine environments and from diverse global samplings. In particular, we analysed three major metabarcoding projects (figure 1): (1) BioMarks, with benthic and pelagic samples from European coastal areas (biomarks.eu), (2) Tara Oceans, with pelagic samplings from all over the world (oceans.taraexpeditions.org) and (3) a deep-sea project (hereafter DeepSea), with benthic samples from great depths (more than 3000 m) in both North Pacific and North Atlantic Oceans [26].

**Figure 2.**
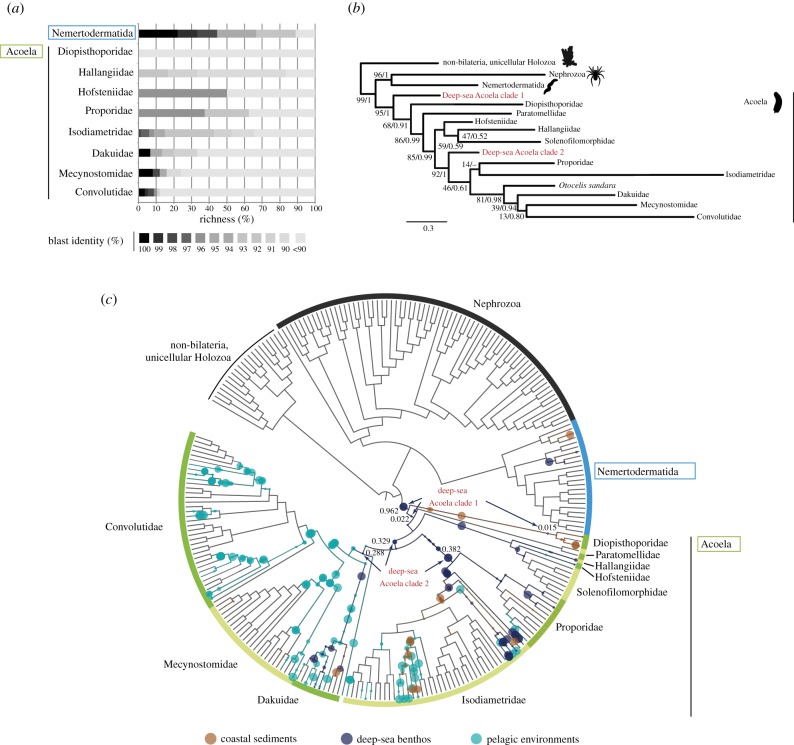
Molecular novelty in Acoelomorpha. (*a*) Blast identity of 101 acoelomorph OTUs against the Acoelomorpha 18S GenBank database in known well-described families [27]. Note the high percentage of richness with low sequence similarity to Acoela. (*b*) Maximum-likelihood tree inferred from 101 Acoelomorpha OTUs and RefTree GenBank sequences (see Materials and Methods). Nodal support indicates 1000 ML bootstrap replicates and posterior probabilities. Coloured OTUs represent novel molecular linages within Acoela. (*c*) Phylogenetic placement of Acoelomorpha OTUs using pplacer software (see Materials and Methods). Our data show that a large number of OTUs cannot be assigned to a sequenced acoelomorph species in the reference tree. Deep sea clades are shown in red, with arrows pointing out to the phylogenetic placements. LWR (likelihood weight ratio) of each placement is displayed near each node.

We found a total of 101 Xenacoelomorpha environmental OTUs (figure 1 and Material and Methods; see electronic supplementary material, S1 and S2 for raw data). Of those, 97 OTUs corresponded to Acoela and four to Nemertodermatida. We did not recover any *Xenoturbella* OTUs. Interestingly, a high percentage (74%) of those sequences show a low blast identity (less than 90%) against the Acoelomorpha 18S rDNA data present in NCBI (figure 2*a*). This indicates that most of the sequences we recovered are molecularly quite different to the acoelomorphs sequenced so far, even though extensive sampling efforts have been undertaken for acoelomorphs in the last decade [10–12,27].

In order to relate the Acoelomorpha novelty with their phylogeny, we performed a phylogenetic placement of all our OTUs against our Acoelomorpha reference tree using pplacer (see Material and Methods). The more internally an OTU is located by pplacer in the tree, the more molecularly different this OTU is compared with the known reference database. Interestingly, more than half of the acoelomorph OTUs (68%) appeared phylogenetically located in internal rather than external nodes of the Acoelomorpha tree (figure 2*c*). Therefore, our data indicate that the genetic diversity of Acoelomorpha is much broader than previously thought.

To identify the exact phylogenetic position of our OTUs, we performed ML and Bayesian inference phylogenetic trees (figure 2*b*; electronic supplementary material, figure S1). Our trees confirmed that some of the new molecular diversity was found in pivotal positions as sister group to major clades. Two OTUs were especially noteworthy, because they probably represent completely new lineages. This is the case of the OTU_DS_13115-11580 (which we name as ‘deep sea Acoela clade 2’), which appears as the sister group of the Crucimusculata group [27]. Even more important is the finding of a new clade (‘deep-sea Acoela clade 1’, from OTU_DS_4335-14605) that represents, with high statistical support, the sister group to the rest of Acoela. This novel acoel clade branches earlier than Diopisthoporidae, an acoel family thought to be the earliest off-shoot and suggested to possess many ancestral characters [27]. Interestingly, both OTUs representing novel clades were found in very deep environments, where the physico-chemical conditions differ from those of shallow coastal areas. While deep sea Acoela 2 was found in fine mud at 4878 m depth in the North Atlantic Ocean, deep sea Acoela clade 1 was found at a depth of 3678 m in the North Pacific Ocean, around 170 km offshore from Monterey Bay, California. This finding suggests that deep benthos is an ideal habitat in which to search for new acoelomorph taxa that may provide important information about the full genomic and morphological diversity of this group. It is perhaps not surprising, then, that the most recently described *Xenoturbella* species were also identified in that habitat [7].

Having identified the most appropriate habitats for sampling of key acoel lineages, we then analysed the full diversity of our OTUs among all samples. These data revealed interesting biogeographic patterns in acoels. For example, some acoel OTUs appear to be cosmopolitan and very abundant in pelagic environments. This is surprising given that only a few acoel species had been described as planktonic [19]. These species have ecological capabilities that distinguish them from sedimentary acoels, such as strong endosymbiont relationships with algae and mixotrophy strategies [19] that could help them to cope with the oligotrophic condition in open marine waters. Thus, our high-throughput analysis indicates that there is a greater complexity in the ecology and lifestyle of acoels than previously suspected (see electronic supplementary material for an extended discussion of the differences between Nemertodermatida and Acoela diversity).

Overall, our data reveal substantial hidden molecular diversity in Acoelomorpha, especially within acoels, than shown in previous morphological studies. In particular, we show that plankton harbours a huge diversity of unsampled acoels, although within known families, while deep-sea sediments have the potential to uncover key novel taxa, including the here reported putative sister group to the rest of acoels. As Hejnol & Pang [8] pointed out, ‘strategic sampling is essential for understanding the evolution of major traits’. We believe that our data could help to design future projects with the specific goal of finding new morphospecies from phylogenetically relevant lineages in which the study of anatomical, morphological and molecular evolution could be carried out.

## Supplementary Material

Supplementary Material: Extended material and methods and analysis of differences between Nemertodermatida and Acoela diversity

## Supplementary Material

Supplementary Figure 1: Maximum-likelihood (ML) tree using 18S GenBank reference sequences and the environmental OTUs identified in this study

## Supplementary Material

Supplementary_Material1.txt (named Final alignment.txt in the latest submission): Alignment of 18S GenBank reference sequences and the environmental OTUs identified in this study

## Supplementary Material

Supplementary_Material2.txt (named Xenacoelomorpha_OTUs_sequences.txt in the latest submission): Fasta file with all Xenacoelomorpha sequences (OTUs) reported in this study

## Data accessibility

Sequences here reported and the phylogenetic alignment are available as electronic supplementary material.

## References

[1] Dunn CW, Giribet G, Edgecombe GD, Hejnol A 2014 Animal phylogeny and its evolutionary implications. Annu. Rev. Ecol. Evol. Syst. 45, 371–395. (10.1146/annurev-ecolsys-120213-091627)

[2] Baguñà J, Riutort M 2004 The dawn of bilaterian animals: the case of acoelomorph flatworms. BioEssays 26, 1046–1057. (10.1002/bies.20113) 15382134

[3] Hejnol A et al. 2009 Assessing the root of bilaterian animals with scalable phylogenomic methods. Proc. R. Soc. B 276, 4261–4270. (10.1098/rspb.2009.0896) PMC2817096 19759036

[4] Cannon JT, Vellutini BC, Smith J, Ronquist F, Jondelius U, Hejnol A 2016 Xenacoelomorpha is the sister group to Nephrozoa. Nature 530, 89–93. (10.1038/nature16520) 26842059

[5] Jondelius U, Ruiz-Trillo I, Baguñà J, Riutort M 2002 The Nemertodermatida are basal bilaterians and not members of the Platyhelminthes. Zool. Scr. 31, 201–215. (10.1046/j.1463-6409.2002.00090.x)

[6] Ruiz-Trillo I, Riutort MJ, Littlewood DT, Herniou EA, Baguña J 1999 Acoel flatworms: earliest extant bilaterian Metazoans, not members of Platyhelminthes. Science 283, 1919–1923. (10.1126/science.283.5409.1919) 10082465

[7] Rouse GW, Wilson NG, Carvajal JI, Vrijenhoek RC 2016 New deep-sea species of *Xenoturbella* and the position of Xenacoelomorpha. Nature 530, 94–97. (10.1038/nature16545) 26842060

[8] Hejnol A, Pang K 2016 Xenoacoelomorpha's significance for understanding bilaterian evolution. Curr. Opin. Genet. Dev. 39, 48–54. (10.1016/j.gde.2016.05.019) 27322587

[9] Haszprunar G 2015 Review of data for a morphological look on Xenacoelomorpha (Bilateria incertae sedis). Org. Divers. Evol. 16, 1–27.

[10] Meyer-Wachsmuth I, Curini Galletti M, Jondelius U 2014 Hyper-cryptic marine meiofauna: species complexes in Nemertodermatida. PLoS ONE 9, e107688 (10.1371/journal.pone.0107688) 25225981 PMC4166464

[11] Zauchner T, Salvenmoser W, Egger B 2015 A cultivable acoel species from the Mediterranean, *Aphanostoma pisae* sp. nov. (Acoela, Acoelomorpha). Zootaxa 3941, 401–413. (10.11646/zootaxa.3941.3.6) 25947519

[12] Curini-Galletti M et al. 2012 Patterns of diversity in soft-bodied meiofauna: dispersal ability and body size matter. PLoS ONE 7, 1–13. (10.1371/journal.pone.0033801) PMC3311549 22457790

[13] Graff LV 1882 Monographie der Turbellarien I. Rhabdocoelida. Leipzig, Germany: Verlag von Wilheilm Engelman: I. I-Ix, 1–442.

[14] Hyman LH 1940 The invertebrates: Protozoa through Ctenophora: *vol. 1*. New York, NY: McGraw-Hill:.

[15] Nakano H, Lundin K, Bourlat SJ, Telford MJ, Funch P, Nyengaard JR, Obst M, Thorndyke MC 2013 *Xenoturbella bocki* exhibits direct development with similarities to Acoelomorpha. Nat. Commun. 4, 1537 (10.1038/ncomms2556) 23443565 PMC3586728

[16] Nastasescu M, Popescu–Marinescu V 2004 Turbellaria spreading within iron gates area existing in benthic and phytophile fauna. Revue Roumaine de Biologie Serie de Biologie Animale 47, 97–10.

[17] Vila-Farré M, Álvarez-Presas M, Achatz JG 2013 First record of *Oligochoerus limnophilus* (Acoela, Acoelomorpha) from British waters. Arx. Miscel·lània Zoològica 11, 153–157.

[18] Ax P, Dörjes J 1966 *Oligochoerus limnophilus* nov. spec. ein kaspisches Faunenelement als erster Süßwasservertreter der Turbellaria Acoela in Flüssen Mitteleuropas. Internationale Revue der Gesamten Hydrobiologie 51, 15–44. (10.1002/iroh.19660510104)

[19] Stoecker D, Swanberg N, Tyler S 1989 Oceanic mixotrophic flatworms. Mar. Ecol. Prog. Ser. 58, 41–51. (10.3354/meps058041)

[20] Katoh K, Standley DM 2013 MAFFT multiple sequence alignment software version 7: improvements in performance and usability. Mol. Biol. Evol. 30, 772–780. (10.1093/molbev/mst010) 23329690 PMC3603318

[21] Stamatakis A 2014 RAxML version 8: a tool for phylogenetic analysis and post-analysis of large phylogenies. Bioinformatics 30, 1312–1313. (10.1093/bioinformatics/btu033) 24451623 PMC3998144

[22] Berger SA, Krompass D, Stamatakis A 2011 Performance, accuracy, and web server for evolutionary placement of short sequence reads under maximum likelihood. Syst. Biol. 60, 291–302. (10.1093/sysbio/syr010) 21436105 PMC3078422

[23] Ronquist F, Huelsenbeck JP 2003 MrBayes 3: Bayesian phylogenetic inference under mixed models. Bioinformatics 19, 1572–1574. (10.1093/bioinformatics/btg180) 12912839

[24] Matsen FA, Kodner RB, Armbrust EV 2010 pplacer: linear time maximum-likelihood and Bayesian phylogenetic placement of sequences onto a fixed reference tree. BMC Bioinform. 11, 538 (10.1186/1471-2105-11-538) PMC3098090 21034504

[25] Camacho C, Coulouris G, Avagyan V, Ma N, Papadopoulos J, Bealer K, Madden TL 2009 BLAST plus: architecture and applications. BMC Bioinform. 10, 1 (10.1186/1471-2105-10-421) PMC2803857 20003500

[26] Bik HM, Sung W, De Ley P, Baldwin JG, Sharma J, Rocha-Olivares A, Thomas WK 2012 Metagenetic community analysis of microbial eukaryotes illuminates biogeographic patterns in deep-sea and shallow water sediments. Mol. Ecol. 21, 1048–1059. (10.1111/j.1365-294X.2011.05297.x) 21985648 PMC3261328

[27] Jondelius U, Wallberg A, Hooge M, Raikova OI 2011 How the worm got its pharynx: phylogeny, classification and bayesian assessment of character evolution in Acoela. Syst. Biol. 60, 845–871. (10.1093/sysbio/syr073) 21828080

