# Supplementary material for: Hidden diversity of Acoelomorpha revealed through metabarcoding
Source: Biol Lett. 2016 Sep;12(9):20160674. doi: 10.1098/rsbl.2016.0674 (PMC5046940; doi:10.1098/rsbl.2016.0674)
Supplement: Supplementary Material: Extended material and methods and analysis of differences between Nemertodermatida and Acoela diversity [file rsbl20160674supp1.docx]

**Biology Letters**

**Hidden diversity of Acoelomorpha revealed through metabarcoding**

Alicia S. Arroyo, David López-Escardó, Colomban de Vargas & Iñaki Ruiz-Trillo

**Supplementary Material:**

1. **Differences between Nemertodermatida and Acoela diversity**
2. **Extended Materials and Methods**
3. **References Supplementary Materials**
4. **Differences in Nemertodermatida and Acoela diversity**

Analysis of the differences between Nemertodematida and Acoela revealed that acoels and nemertodermatids differ from both a phylogenetic and an ecological perspective. Phylogenetically, nemertodermatids OTUs, in contrast to acoels, did not show any putative novelty. Blast identity against Acoelomorpha 18S rDNA GenBank was very high ($\overline{x}=99.52 \left( SD 0.62 \right)$), with most of them placed in external nodes (figure 2A and C). This corroborates morphological studies in which only 9-11 out of 400 described Acoelomorpha species corresponded to nemertodermatids [1,2]. Another distinction is the fact that all four nemertodermatid OTUs appear to be present in sediments, which are already known as a habitat for these flatworms [3]. In contrast, none were found in plankton. Therefore, our data suggest that nemertodermatids are genetically less diverse than acoels. Nevertheless, this observation should be interpreted with caution because the 18S rDNA gene alone is not able to detect the full diversity in nemertodermatids, which also display complex morphological cryptic patterns according to Meyer-Wachsmuth [4]. However, given the phylogenetic closeness and the apparent morphological similarity between acoels and nemertodermatids, the huge difference in molecular diversity is striking.

These differences between acoels and nemertodermatids could be explained by the presence of more complex structures and lifestyles in acoels. These include 1) a more complex nervous system [5], 2) the presence of photoreception organs [6,7], 3) more complex sexual interactions [5,8-12] , and 4) the possibility of mixotrophy and symbiotic relationships with algae [13-15]. Whether all of those differences in body plan and lifestyles could have led to a greater ability of acoels to cope with different habitat constraints remains unclear.

1. **Extended Materials and Methods**

**Acoelomorph OTUs**

Clustered OTUs (Operational Taxonomic Units) were obtained from their corresponding public repositories or directly from the authors. In TaraOceans, swarm-clustered OTUs had been taxonomically assigned using PR2 database [16]. BioMarks OTUs had been clustered at 97% homology with USEARCH [17], but the taxonomic assignation had been done using a manually curated in-home metazoan database based on PR2. In DeepSea, OTUs had been clustered at 99% homology using QIIME [18] and we performed a taxonomic assignation based on our in-home metazoan database. Both BioMarks and DeepSea OTUs were further checked for chimeras against Silva database, using the mothur version of ChimeraSlayer [19].

**Reference tree**

An Acoelomorpha curated tree was first constructed from 255 acoelomorph 18S rDNA GenBank sequences (from herein RefTree). Sequences were aligned using E-INS-I method from MAFFT v7.271 alignment software [20] and then manually checked and trimmed with Geneious v8.0.5 [21]. Non-homologous highly variable positions were removed from the alignment leading to a final length of 1908 nucleotide positions. Finally, the maximum likelihood (ML) tree was built by using RAxML v8.0.0 software [22] considering a GTR-GAMMA substitution model. Nodal support was obtained through 1000 bootstrap replicates (alignment of all sequences are available in Supp. Material 1).

We placed the OTUs short reads using RAxML-EPA algorithm [23]. We removed from further analyses all sequences that either 1) did not align in their proper region, or 2) were not placed within Acoelomorpha clade, or 3) had extremely long branches, making the rate of nucleotide substitutions greater than 1. Final OTUs were then selected if their abundances were greater than 10 reads in order to be sure that the OTUs were representative enough. DeepSea forward and reverse clustered OTUs were considered as one if both had a percentage of pairwise identity greater than 99% with MUSCLE alignment software [24].

**Phylogenetic analysis**

A ML tree of the 18S rDNA gene using both the RefTree sequences and our OTUs was inferred using RAxML v8.0.0 software [23], under a GTR-GAMMA model of evolution. Nodal support was obtained by 1000 ML bootstrap replicates. A Bayesian tree was built using MrBayes v3.2.6 [25,26] after 30 million generations, using a GTR model of evolution with gamma-distributed rate variation across sites with four discrete categories and a proportion of invariable sites –pinv- (GTR+I+G). Using Tracer v1.6.0 [27] we observed that there was no toxic interaction between alpha shape parameter and pinv. Then, we checked for convergence diagnosis, verifying that all the parameter estimates have converged. The concatenated tree from both ML and Bayesian inference is available in Suppl. Figure 1.

Pplacer package v1.1 [28] was used to perform a phylogenetic placement of the OTUs into the Acoelomorpha RefTree and posterior visualization was done using iTOL web-tool [29].

Novelty blast percentages were obtained running a blastn 2.2.31 [30] against our curated Acoelomorpha-GenBank database. Blast hits were, then, classified into percentage categories using an in-home script.

1. **References Materials and Methods**
2. Sterrer W. 1998. New and known Nemertodermatida (Platyhelminthes-Acoelomorpha): A revision. *Belgian J. Zool*. **128**,55–92.
3. Jondelius U, Ruiz-Trillo I, Baguñà J, Riutort M. 2002. The Nemertodermatida are basal bilaterians and not members of the Platyhelminthes. *Zool. Scr.* **31**,201–215.
4. Haszprunar G. 2015. Review of data for a morphological look on Xenacoelomorpha (Bilateria incertae sedis). *Org. Divers. Evol.* **16**, 1–27.
5. Meyer-Wachsmuth I, Curini Galletti M, Jondelius U. 2014. Hyper-cryptic marine meiofauna: Species complexes in Nemertodermatida. *PLoS One* **9**:e107688.
6. Achatz JG, Martinez P. 2012. The nervous system of *Isodiametra pulchra* (Acoela) with a discussion on the neuroanatomy of the Xenacoelomorpha and its evolutionary implications. *Front. Zool*. **9**:27.
7. Yamasu T. 1991. Fine structure and function of ocelli and sagittocysts of acoel flatworms. *Hydrobiologia* **227**,273–282.
8. Hejnol A, Obst M, Stamatakis A, Ott M, Rouse GW, Edgecombe GD, Martinez P, Baguñà J, Bailly X, Jondelius U, et al. 2009. Assessing the root of bilaterian animals with scalable phylogenomic methods. *Proc. Biol. Sci.* **276**,4261–4270.
9. Hyman LH. 1937. Reproductive system and copulation in *Amphiscolops langerhansi* (Turbellaria acoela). *Biological Bulletin Wood's Hole* **72**,319–326.
10. Costello HM, Costello DP. 1938. Copulation in the Acoelous Turbellarian *Polychoerus carmelensis*. *Biological Bulletin Wood's Hole* **75**,85–98.
11. Westblad E. 1946. Studien über skandinavische Turbellaria Acoela. *Arkiv för Zoologi*, **38**,1-56.
12. Apelt G. 1969. Fortpflanzungsbiologie, Entwicklungszyklen und ver-gleichende Frühentwicklung acoeler Turbellarien. *Marine Biology (Berlin)* **4**,267–325.
13. Bush L. 1975. Biology of *Neochilda fusca* n. gen., n. sp. from the Northeastern Coast of the United States (Platyhelminthes: Turbellaria). *Biol. Bull.* **148**,35–48.
14. Löhner L, Micoletzky H. 1911. Ueber zwei neue pelagische Acoelen des GoIfes von Triest (*Convoluta pelagica* und *Monochoerus illardatus*). *Z. Wiss. Zool*. **98**,381-42.
15. Dörjes J. 1970. *Haplodiscus bocki* spec. nov., eine neue pelagische Turbellarie der Ordnung Acoela von der Molukken-See mit einer Diskussion der Gattung. *Ark. Zool.* **23**,255-26.
16. Stoecker D, Swanberg N, Tyler S. 1989. Oceanic mixotrophic flatworms. *Mar. Ecol. Prog. Ser.* **58**,41–51.
17. de Vargas C, Audic S, Henry N, Decelle J, Mahé F, Logares R, Lara E, Berney C, Le Bescot N, Probert I, et al. 2015. Eukaryotic plankton diversity in the sunlit ocean. *Science*, **348**:1261605-1-11.
18. Massana R, Gobet A, Audic S, Bass D, Bittner L, Boutte C, Chambouvet A, Christen R, Claverie JM, Decelle J, et al. 2015. Marine protist diversity in European coastal waters and sediments as revealed by high-throughput sequencing. *Environ. Microbiol.* **17**,4035–4049.
19. Bik HM, Sung W, De Ley P, Baldwin JG, Sharma J, Rocha-Olivares A, Thomas WK. 2012. Metagenetic community analysis of microbial eukaryotes illuminates biogeographic patterns in deep-sea and shallow water sediments. *Mol. Ecol.* **21**,1048–1059.
20. Schloss PD, Westcott SL, Ryabin T, Hall JR, Hartmann M, Hollister EB, Lesniewski RA, Oakley BB, Parks DH, Robinson CJ, et al. 2009. Introducing mothur: Open-source, platform-independent, community-supported software for describing and comparing microbial communities. *Appl. Environ. Microbiol*. **75**,7537–7541.
21. Katoh K, Standley DM. 2013. MAFFT multiple sequence alignment software version 7: Improvements in performance and usability. *Mol. Biol. Evol.* **30**,772–780.
22. Kearse M, Moir R, Wilson A, Stones-Havas S, Cheung M, Sturrock S, Buxton S, Cooper A, Markowitz S, Duran C, et al. 2012. Geneious Basic: An integrated and extendable desktop software platform for the organization and analysis of sequence data. *Bioinformatics* **28**,1647–1649.
23. Stamatakis A. 2014. RAxML version 8: A tool for phylogenetic analysis and post-analysis of large phylogenies. *Bioinformatics* **30**,1312–1313.
24. Berger SA, Krompass D, Stamatakis A. 2011. Performance, accuracy, and web server for evolutionary placement of short sequence reads under maximum likelihood. *Syst. Biol.* **60**,291–302.
25. Edgar RC. 2004. MUSCLE: Multiple sequence alignment with high accuracy and high throughput. *Nucleic Acids Res*. **32**,1792–1797.
26. Ronquist F, Huelsenbeck JP. 2003. MrBayes 3: Bayesian phylogenetic inference under mixed models. *Bioinformatics* **19**,1572–1574.
27. Altekar G, Dwarkadas S, Huelsenbeck JP, Ronquist F. 2004. Parallel Metropolis-coupled Markov chain Monte Carlo for Bayesian phylogenetic inference. *Bioinformatics* **20**,407-415.
28. Rambaut A, Suchard MA, Xie D, Drummond AJ. 2014. Tracer v1.6, available from <http://beast.bio.ed.ac.uk/Tracer>.
29. Matsen FA, Kodner RB, Armbrust EV. 2010. pplacer: linear time maximum-likelihood and Bayesian phylogenetic placement of sequences onto a fixed reference tree. *BMC Bioinformatics* **11**,538.
30. Letunic I, Bork P. 2007. Interactive Tree Of Life (iTOL): An online tool for phylogenetic tree display and annotation. *Bioinformatics* **23**,127–128.
31. Camacho C, Coulouris G, Avagyan V, Ma N, Papadopoulos J, Bealer K, Madden TL. 2009. BLAST plus: architecture and applications. *BMC Bioinformatics* **10**:1.
